# Consensus of potential modifiable prognostic factors for persistent pain after a first episode of nonspecific idiopathic, non-traumatic neck pain: results of nominal group and Delphi technique approach

**DOI:** 10.1186/s12891-020-03682-8

**Published:** 2020-10-07

**Authors:** Martine Verwoerd, Harriet Wittink, Francois Maissan, Rob Smeets

**Affiliations:** 1 Research Group Lifestyle and Health, Utrecht University of Applied Sciences, Heidelberglaan 7, Utrecht, the Netherlands; 2 Research Group Lifestyle and Health, Utrecht University of Applied Sciences, Heidelberglaan 7, Utrecht, the Netherlands; 3 Department of Rehabilitation Medicine, Research School CAPHRI, CIR Rehabilitation, Maastricht University, Eindhoven, The Netherlands

**Keywords:** Prognostic factors, Chronic neck pain, Idiopathic neck pain, Prognostic factors, Delphi survey

## Abstract

**Background:**

Identify and establish consensus regarding potential prognostic factors for the development of chronic pain after a first episode of idiopathic, non-traumatic neck pain.

**Design:**

This study used two consensus group methods: a modified Nominal Group (m-NGT) and a Delphi Technique.

**Methods:**

The goal of the m-NGT was to obtain and categorize a list of potential modifiable prognostic factors. These factors were presented to a multidisciplinary panel in a two-round Delphi survey, which was conducted between November 2018 and January 2020. The participants were asked whether factors identified are of prognostic value, whether these factors are modifiable, and how to measure these factors in clinical practice. Consensus was a priori defined as 70% agreement among participants.

**Results:**

Eighty-four factors were identified and grouped into seven categories during the expert meeting using the modified NGT. A workgroup reduced the list to 47 factors and grouped them into 12 categories. Of these factors, 26 were found to be potentially prognostic for chronification of neck pain (> 70% agreement). Twenty-one out of these 26 factors were found to be potentially modifiable by physiotherapists based on a two-round Delphi survey.

**Conclusion:**

Based on an expert meeting (m-NGT) and a two-round Delphi survey, our study documents consensus (> 70%) on 26 prognostic factors. Twenty-one out of these 26 factors were found to be modifiable, and most factors were psychological in nature.

## Background

Commonly it is assumed that most episodes of acute idiopathic neck pain will resolve with or without treatment. However, Childs et al. (2008) argue that rates of persistent neck pain are substantial [1]. It is suggested that the prognosis of acute neck pain is worse than currently recognized [2]. Twenty-four to 37% of individuals who experience neck pain will report persistent problems for at least 12 months [3]. In the Netherlands, neck pain is the most prevalent disorder presented at physiotherapy practices [4].

The reported effect of physiotherapy treatment of chronic musculoskeletal pain is, at best, only moderate [5–7]. Prevention of chronicity must occur in the (sub)acute phase of musculoskeletal pain. Knowledge of prognostic, potentially modifiable factors can help health care providers to improve clinical decision-making and is a likely key in combatting chronification of idiopathic neck pain.

A recent systematic review showed limited evidence to support prognostic factors that are associated with pain or perceived non-recovery up until one year after the onset of neck pain [8]. The quality of the available evidence was graded as low to very low and included only a few modifiable factors. Psychosocial factors as passive coping, catastrophizing, fear-avoidance beliefs, depressive symptoms, distress, and anxiety are potentially modifiable factors that were found to be associated with chronic neck pain, whiplash related neck pain, and low back pain [9–19]. These findings concern other subgroups of musculoskeletal pain, and can therefore not be generalized to patients with idiopathic nonspecific, non-traumatic, acute or subacute neck pain.

It is known that neurophysiological changes in chronification of pain are modulated by psychosocial factors [20]. It is therefore surprising that prior research on chronification of idiopathic nonspecific, non-traumatic, acute- or subacute musculoskeletal neck pain is frequently done from a biomedical perspective only. At this stage, it is still unclear which factors are potentially prognostic and modifiable by physiotherapists in this subgroup. Starting this study with a wider view (i.e. biopsychosocial framework), seems to be important.

### Purpose of the study

To establish consensus regarding potential prognostic factors for the development of chronic pain after a first episode of idiopathic, non-traumatic neck pain and whether experts consider these factors as modifiable by physiotherapy interventions, by using a modified Nominal Group Technique (m-NGT) and a Delphi survey instrument.

## Method

### Study design

**Fig. 1:**
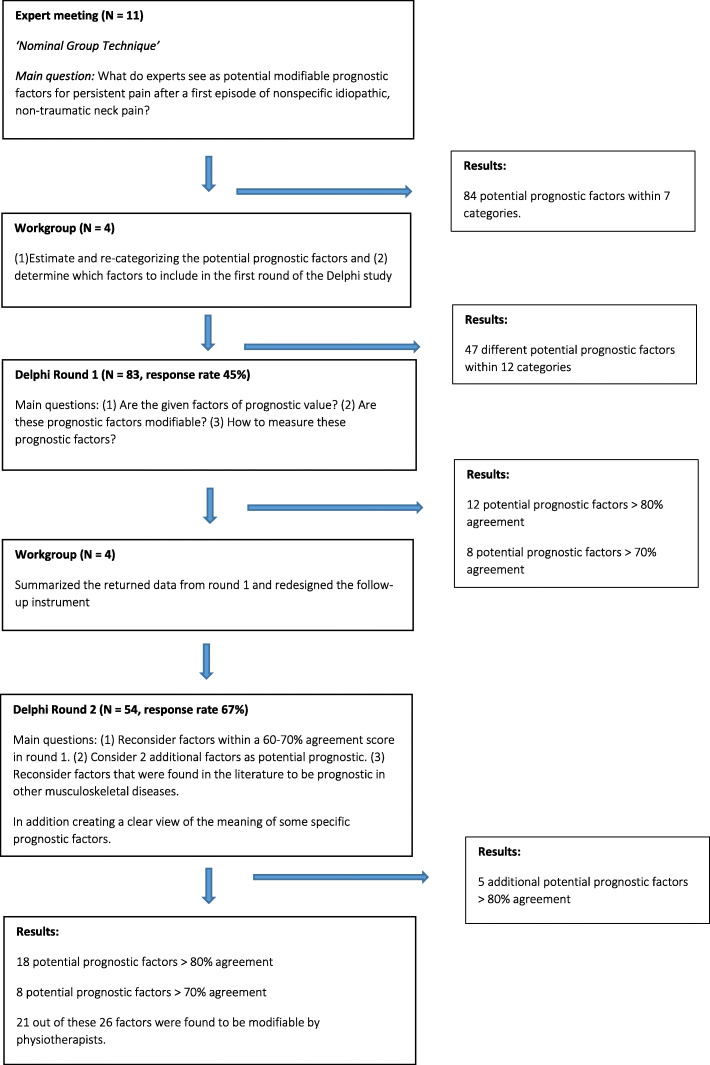
Flow chart study

This study used two consensus group methods; a m-NGT and Delphi Technique [21, 22]. The study was conducted between November 2018 and January 2020. Ethical approval and consent to participate in our Delphi and expert meeting was not required based on the Dutch- Medical Research Involving Human Subjects Act (WMO). Figure 1 presents the flow-chart of our study.

### Expert meeting

We conducted a m-NGT meeting. In general, NGT uses a highly structured meeting to gather information from relevant experts about a pre-specified topic with a focus on a single goal [21]. This technique comprises four stages: silent generation, round robin, clarification and ranking [23]. The goal in this study was to identify prognostic factors for persistent pain after a first episode of idiopathic, non-specific neck pain to include in a Delphi for consensus. Therefore, we did not complete the ranking stage as is described in a classic NGT but categorized the prognostic factors.

#### Selection of participants

A NGT usually involving 5–12 experts in the field [22]. Our m-NGT group consisted of 11 experts plus two members of the research team. The two members of the research team facilitated the process and were specifically instructed not to influence the participants [22]. Being an expert entails the acquisition of experience or knowledge of a particular topic [24]. The experts were either working in (1) specialized physiotherapy clinics for nonspecific neck pain patients, and/or (2) working in neck pain research, and/or (3) were academic teachers with a special focus on the neck. To reach a heterogeneous group, we have taken into account a reliable distribution in credentials, occupation at the time of the study, and specialization. The participants of the expert group meeting were selected from the ‘Pain Community’ of the University of Applied Sciences in Utrecht and supplemented by experts from the national network of our research group.

#### Procedure

Before the expert meeting took place, each participant received a digital file consisting of (i) a summary of the results of a recently performed systematic review on prognostic factors for persistent neck pain [8], and (ii) an introduction to our consensus study. Knowledge of these results was the starting point of our expert meeting. The expert meeting followed 4 steps:

1. Introduction of the structure of the meeting and the main question of the meeting: ‘*What do experts see as potential modifiable prognostic factors for persistent pain after a first episode of nonspecific idiopathic, non-traumatic neck pain?’;*
2. Brainstorming and writing down ideas about potential modifiable prognostic factors by each participant (10 min);
3. Presenting, operationalizing and generating more ideas in groups of 2 to 3 participants (this stage takes as much time as needed until no new ideas are forthcoming [25]);
4. Presenting the operationalized ideas to all experts, followed by a group discussion (30 min). Towards the end of the discussion the prognostic factors were categorized.

#### Data analysis

The data was analyzed by a workgroup of four research and clinical experts (HW, MV, FM, ER). The analysis included (1) assessing for overlapping factors (2) re-categorizing the biomedical prognostic factors, and (3) re-categorizing the psychological factors. For re-categorizing psychological factors, an expert in physiotherapy in mental health and psychology was also consulted.

### Delphi survey

#### Selection of participants

Participants were selected via purposive sampling to ensure that each participant had in-depth knowledge of the problem.

Our sampling started at an Dutch−/ Belgium multidisciplinary research consortium ‘pain in motion’ that focuses on improving the understanding of biopsychosocial mechanisms of pain. Then a search in the PubMed database was performed for the identification of participants across the world with diverse backgrounds to guarantee an international base of knowledge. Experts were eligible to participate if (1) they were clinicians with a large experience in the specific area, and/or (2) they (co)authored at least two peer-reviewed publications in the field of nonspecific neck pain and physiotherapy.

An invitation to participate was sent to 185 eligible candidates.

#### Procedure

We conducted a two-round Delphi survey. The factors included in the Delphi survey were taken from our systematic review and the expert meeting, as described earlier [8]. Generating data by other qualitative studies for the first round of a Delphi questionnaire is a common and widely accepted method [26–28].

We sent a digital questionnaire to survey participants in April 2019. The survey contained a letter introducing the study, an invitation to participate, and instructions for completing the questionnaire. If the questionnaire was not returned within 3 weeks of postage, a reminder email was sent after 3 and 5 weeks. Only questionnaires received up to 6 weeks after the first mailing were included in the analysis.

In the first round of the Delphi survey, participants were asked to answer questions in three subsections (see Additional file 1). First, indicate whether the given factors are of prognostic value; second, indicate whether these factors are modifiable or not; and third indicate how to measure these factors. Each subsection also allowed for open commenting. In addition, we asked the participants explicitly to comment on the way of categorizing the psychological factors. Only participants who considered a factor of prognostic value had to answer the questions in subsections two and three.

Although there is no official guideline on optimal consensus, the minimum level of agreement was set at 70%, as suggested in current literature [29–32].

The workgroup (MV, HW, FM, RS) summarized the survey data of round 1 and designed a follow-up questionnaire to be surveyed in the second round (see Additional file 1). The factors on which consensus was reached were not questioned in the second round.

We included the following factors in our second round questionnaire; (i) a prognostic modifiable factor with a 60–70% agreement score (to avoid false-negative findings), (ii) prognostic factors added by participants in the first round, and (iii) factors that did not reach a sufficient agreement score in the first round, though they were found of prognostic value for other musculoskeletal diseases in the literature. All other factors with a below 60% agreement score were excluded.

In case there was ambiguity about the meaning of specific factors added by participants, the participants were asked to clarify these factors in the second-round questionnaire.

The participants of our Delphi survey were mainly experts in musculoskeletal (neck) pain, but not in measurement tools. Therefore, we only used the first Delphi round to get an indication of how to measure these potentially prognostic factors in research and practice, and not to reach consensus.

## Results

### Expert meeting

Table 1 describes the characteristics of the participants of our expert meeting. Our 11 professionals indicated 84 factors to be prognostic for chronification of neck pain. They categorized them into 7 categories; communication, social support, work-related, pain related, lifestyle, biomedical/ biomechanical, and psychological (including thoughts, feelings and behavior).

**Table 1:** Demographics of participants at the expert meeting (*n* = 11)

**Gender**	Male = 8
	Female = 3
**Credentials**	PhD = 1
	PhD student = 2
	MSc = 6
	BSc = 2
Occupation at the time of the study*	Academic researcher = 3
	Academic teacher = 5
	Active practicing musculoskeletal PT = 8
**Specialization**	Orthopedic Manual PT = 2
	PT in Mental Health = 6
	Medical doctor = 1
	Psychologist = 1
	Regular PT = 1

* A number of participants have a dual function. *Abbreviations*: *PhD* Doctor of Philosophy, *MSc* Master of Science, *BSc* Bachelor of Science, *PT* Physiotherapist

### Workgroup

Our workgroup (MV, HW, FM, ER) and our consulted expert analyzed and grouped the 84 potential prognostic factors into 47 factors within 12 categories; social demographic, work-related, symptoms, prior conditions, general factors, cognition, emotions, behavior, perceptions, motivation, vulnerability and remaining (health care provider attitude and therapeutic relation) factors. We did so because (i) there was a strong overlap between a number of these 84 factors, and (ii) the 7 categories were too broad and therefore not specific enough. Table 2 presents all factors and categories.

**Table 2:** Consensus agreement of prognostic factors Delphi-survey

Prognostic factors	Number of participants per factor Round 1	Percentage agreement (yes) Round 1	Number of participants per factor Round 2	Percentage Agreement (yes) Round 2
**Social demographic**				
Gender	80	56.25%	–	–
Age	80	65%	–	–
Social class	80	56.25%	–	–
Education level	80	66.25%	–	–
Marital status	80	11.24%	–	–
**Work-related factors**				
Employment status	80	53.75%	–	–
Happiness in work**	**80**	**86.25%**	**–**	**–**
Physical work	80	53.75%	–	–
**Symptoms**				
Pain intensity at baseline**	80	65%	–	**87.50%**
Duration of the neck pain*	**80**	**72.50%**	**–**	**–**
*Disturbed sleep due to neck pain*	80	60%	–	–
Reported pain in different body regions*	**80**	**78.75%**	–	–
High severity of disability	80	51.25%	–	–
High severity of experienced disability**	80	65%	48	**91.67%**
Cervical mobility	80	12.50%	–	–
Thoracic mobility	80	10%	–	–
Cervical motor control	80	25%	–	–
Posture	80	13.75%	–	–
Radiating pain below elbow	80	30%	–	–
Accompanying headache	80	36.25%	–	–
Dizziness	80	18.75%	–	–
Pressure sensitivity neck musculature	80	25%	–	–
**Prior conditions**				
Neck pain before**	**70**	**92.86%**	–	–
History of musculoskeletal pain*	**70**	**72.86%**	–	–
**General factors**				
Physical inactivity**	**71**	**90.14%**	–	–
Unhealthy lifestyle (smoking, alcohol, eating etc.)*	**71**	**76.06%**	–	–
Sleep quality*	**71**	**73.24%**	–	–
**Cognition**				
Somatization**	74	62.16%	48	**89.58%**
Catastrophizing**	**74**	**87.84%**	–	–
Locus of control	74	59.46%	–	–
Acceptance of illness	74	52.70%	–	–
Illness beliefs about recovery**	**74**	**83.78%**	–	–
Treatment beliefs*	**74**	**70.27%**	–	–
**Emotions**				
Depression**	**72**	**87.50%**	–	–
Kinesiophobia**	**72**	**86.11%**	–	–
Distress*	**72**	**72.22%**	–	–
Anger	72	43.06%	–	–
Injustice	72	40.28%	–	–
**Behavior**				
Coping**	**70**	**95.71%**	–	–
**Perceptions**				
Illness beliefs about pain identity**	**56**	**89.29%**	–	–
Hypervigilance *	**56**	**76.79%**	–	–
**Motivation**				
Purposeful behavior**	32	**90.63%**	–	–
**Vulnerability**				
Limited health literacy **	62	62.90%	48	**87.50%**
Limited self-regulation	62	50%	–	–
Limited self-efficacy**	**62**	**88.71%**	–	–
**Remaining factors**				
Health care provider attitude (biomedical/biopsychosocial)**	**65**	**90.77%**	–	–
Therapeutic relation**	**65**	**84.62%**	–	–
**Additional factors round 2**				
Orofacial pain	–	–	40	65%
Potential to self-modify posture**	–	–	**40**	**82.50%**

Factors with an agreement > 70% shown in bold (* > 70% agreement. ** > 80% agreement). Factors shown in italics were found not unambiguous and were asked to clarify in the second-round questionnaire

### Delphi survey

#### First round

The first-round questionnaire was returned by 83 participants (response rate 45%). The most common professional backgrounds of the participants were researchers with a specialization in neck or chronic pain and orthopedic manual therapists. Table 3 describes the characteristics of the participants in round 1 and 2 of our Delphi survey.

**Table 3:** Demographics of participants at the Delphi-survey

	Delphi-participants in Round 1 (185 eligible candidates invited, response ***N*** = 83, response rate 45%)	Delphi-participants Round 2 (81 participants invited*, response ***N*** = 54, response rate 67%)
**Gender**	Male = 56%	Male = 59%
	Female = 44%	Female = 41%
**Country of residence**	The Netherlands = 30	The Netherlands = 24
	Belgium = 18	Belgium = 10
	Saudi Arabia = 2	Saudi Arabia = 1
	Canada = 5	Canada = 2
	Australia = 3	Australia = 2
	Sweden = 2	Sweden = 1
	Switzerland = 3	Switzerland = 3
	Brazil = 1	France = 1
	France = 1	UK = 2
	UK = 2	South – Africa = 1
	South – Africa = 1	Italy = 1
	Italy = 2	Thailand = 1
	Thailand = 1	Spain = 1
	Spain = 1	USA = 1
	Norway = 1	Portugal = 1
	USA = 1	New-Zealand = 1
	Portugal = 2	Denmark = 1
	New-Zealand = 1	
	Denmark = 1	
	Not given = 2	
**Specialization**	Researcher, specialization neck or chronic pain = 42	Researcher, specialization neck or chronic pain = 26
	Physiotherapist = 18	Physiotherapist = 14
	Physiotherapist in Mental Health = 3	Physiotherapist in Mental Health = 2
	Orthopedic Manual physiotherapist = 10	Orthopedic Manual physiotherapist = 7
	Psychologist = 1	Epidemiologist = 6
	Epidemiologist = 8	
	Not given = 1	

*Two participants did not leave their email address, therefore we could only invite 81 participants instead of the 83 responders in the first round

Eight of the 47 potential prognostic factors achieved over 70% agreement, and twelve factors achieved over 80% agreement. Two potentially prognostic factors were also added by participants: orofacial pain and the potential to self-modify posture during work. There was only one participant who comment on the way we categorized our psychological factors. Based on this comment, we did not changed our categories.

#### Second round

The second-round questionnaire was sent to all participants of the first round who submitted answers. The second-round questionnaire was returned by 54 participants (response rate 67%). Lack of participation was not associated with a geographic area or professional background.

All the potential prognostic factors to reconsider in the second round; pain intensity at baseline, high severity of experienced disability, somatization, and limited health literacy, now reached consensus (> 80%). The additional factors, orofacial pain and potential to self-modify posture, reached a 65% and 82,5% agreement score, respectively.

We found 26 factors to be potentially prognostic fordeveloping chronic neck pain. These factors can be divided into the following categories: work-related factors, symptoms, prior conditions, general factors, cognition, emotions, behavior, perceptions, motivation, vulnerability, and remaining factors. Table 2 describes the consensus agreement of prognostic value of the prognostic factors and Table 4 describes the consensus agreement of the modifiability of the 26 potentially prognostic factors.

**Table 4:** Consensus agreement of prognostic factors and modifiability Delphi survey

Potential prognostic factors	Modifiable
**Work related factors**	
Happiness in work	X
Potential to self-modify posture during work	X*
**Symptoms**	
Pain intensity at baseline	–
High severity of experienced disability	X**
Duration of the neck pain	–
Reported pain in different body regions	–
**Prior conditions**	
Neck pain before	–
History of musculoskeletal pain	–
**General factors**	
Physical inactivity	X**
Unhealthy lifestyle	X*
Sleep quality	X*
**Cognition**	
Somatization	X**
Catastrophizing	X**
Illness beliefs about recovery	X**
Treatment beliefs	X**
**Emotions**	
Depression	X
Kinesiophobia	X**
Distress	X**
**Behavior**	
Coping	X**
**Perceptions**	
Illness beliefs about pain identity	X**
Hypervigilance	X**
**Motivation**	
Purposeful behavior	X*
**Vulnerability**	
Limited health literacy	X*
Limited self-efficacy	X**
**Remaining factors**	
Health care provider attitude	X**
Therapeutic relations	X**

X Factors with an agreement score on modifiability > 70%, X* > 80% agreement, X** > 90% agreement. – Not relevant to ask for modifiability or < 70% agreement. Only the participants who considered these factors of prognostic value had to vote for modifiability

The workgroup concluded that the factors *bad sleep quality* and *happiness at work* are ambiguous. For this reason, the workgroup decided to perform a topical survey to get a clear view of the meaning of these factors. We asked the participants in the second-round to describe in a few sentences (1) what they consider to be ‘bad sleep quality’ and how they would measure this factor in practice, and (2) what they think we measure when we ask patients the following question: *‘On a numeric rating scale from 0 to 10, how satisfied are you with your work? (0 = not satisfied at all, 10 = totally satisfied).*

Regarding sleep quality, seven themes were often mentioned: waking up several times per night (52% of the 48 participants who answered these additional questions), waking up unrefreshed (38%), sleep duration or not enough hours (< 6 h) (35%), difficulties falling asleep (31%), not spending an appropriate amount of time in each of the sleeping phases (15%) and waking up early (8%).

Regarding happiness at work, most the participants reported: *“it is a very broad question”* and “*satisfaction with work is not equivalent or the same construct as happiness*”. The participants indicated a total of 30 themes covered in the concept “happiness at work” (e.g. work-related stress, salary aspects, success, balance life/work and the content of work).

In conclusion, both the prognostic factor ‘sleep quality’ and ‘happiness at work’ are covering different concepts, and must, therefore, be measured in more detail.

## Discussion

### Main findings

Following an expert meeting (m-NGT) and a two-round Delphi survey, the expert panel reached consensus (> 70%) on 26 factors to be potentially prognostic for developing chronic neck pain: pain intensity at baseline, happiness in work, high severity of experienced disability, duration of neck pain, reported pain in different body regions, neck pain before, history of musculoskeletal pain, physical inactivity, limited health literacy, unhealthy lifestyle, sleep quality, catastrophizing, illness beliefs about recovery, pain identity and treatment, depression, kinesiophobia, distress, coping, hypervigilance, purposeful behavior, potential to self-modify posture, somatization, limited self-efficacy, health care provider attitude and therapeutic relations. The experts participating in the Delphi found 21 out of these 26 factors to be modifiable by physiotherapists.

### Comparison with previous studies

The results of this study are in line with other prognostic research in musculoskeletal pain. In particular, psychological factors appear of important prognostic value. Psychological stress, fear avoidance beliefs, and catastrophizing were found to be associated with chronic idiopathic, non-traumatic neck [15–17]. Whereas depressive symptoms, coping, distress and catastrophizing were found to be prognostic for chronification of low back pain [11–14]. The findings of these studies cannot be simply generalized to patients with idiopathic nonspecific, non-traumatic, acute or subacute neck pain because these factors have never been properly investigated in this population.

### Strengths and limitations methodology

We conducted two modified consensus methods to answer our research question. Researchers often begin with a local NGT to generate items that are later used in an international Delphi survey. A classic Delphi survey and the NGT Technique follow a prescribed set of procedures that reflect both behavioral and statistical processes [21, 33]. We conducted modified NGT and Delphi techniques, as research suggests that it is important to move away from the use of labels and move toward a comprehensive description of the steps taken in a specific study. We followed a prescribed method on our m-NGT and Delphi to maintain the balanced participation of our participants and the consideration of different perspectives during the process.

Limitations of the NGT method is the potential for dominant participants to unduly influence the group [22]. However, in our study, this was not the case. Ranking the generated ideas is one of the key stages in an NGT. Since our preliminary aim was to explore potential prognostic factors for an international Delphi, we considered the ranking stage not applicable [22].

In order to maintain the rigor of a Delphi technique, a response rate of 70% of invited participants is recommended. Although we did not reach this rate, in neither round was there a lack of participation from a select group based on professional background or geographic area, thus excluding non-response bias.

There is a wide variation in numbers of participants in Delphi studies, according to the scope of the problem and resources available. Although there is little empirical evidence on the effect of the number of participants on the reliability or validity of consensus processes, Murphy et al. (1998) suggest that the reliability of a composite judgement increases in the number of judges [33, 34]. Given the large number of participants and the mix of professional backgrounds involved in both rounds, we assert the sample was representative for a valid outcome of this study.

An important strength of our study is that we used purposive sampling in our m-NGT and Delphi. It is suggested that a heterogeneous group produces a higher proportion of high quality, highly acceptable solutions or recommendations than homogeneous group [23]. In our Delphi study, geographic heterogeneity was not reached. However, heterogeneity was reached in credentials, clinical experience, scientific expertise, specialization and occupation. Our research goal was to generate input for our prognostic study that is explicitly relevant for clinicians. Therefore, we deem the inclusion of both researchers and clinicians in our m-NGT and Delphi study as particularly representative for clinicians, our main focus group.

The first round of our Delphi questionnaire was structured and did not provide the possibility of much open response. It has commonly been assumed that open-ended questions would give the participant the freedom to elaborate on the topic under investigation and may increase the richness of the data collected. However, our first round was based on our systematic review, m-NGT and workgroup meetings, and therefore we believed that a large number of open-ended questions was not necessary. Nevertheless, the role of subjectivity of items supplied by the researchers in the first round could still be questioned.

### Interpretation of findings

Some of our findings must be interpreted with caution because they are likely an overestimation of the degree of consensus. For example, in the second Delphi round we found remarkable high agreement scores (87,5% to 91,7%) for some factors. There are several reasons for this. First, as it is common in Delphi studies, participants had the opportunity to revise their opinion on prognostic factors that did not reach consensus in the first place. While this is usually done for all factors that failed to reach consensus, participants only had to reconsider factors with an original agreement score between 60 to 70% [25]. Second, the high agreement scores might be a result of participants with minority opinions dropping out [35]. Third, participants might have become fatigued of an additional round and agreed to end the process [27].

Unanimous agreement scores were found on the modifiability of some potentially prognostic factors. A reason could be that we only discussed the modifiability with the participants who found these factors to be prognostic. These agreement scores are based on a much lower number of participants compared to the scores for prognostic value. Besides, it is likely that participants who did not agree on the prognostic value of these factors also graded these factors as not modifiable.

### Clinical message and future directions

Twelve out of 26 of our potential prognostic factors and six out of 13 categories are of psychological nature, and hence, are either likely highly correlated [36] and/or do likely have (a) common underlying, or at least partly overlapping construct(s). This may result in different interpretations of these factors/categories across participants, potentially biasing the results of our study. In consequence, we call for greater clarity on the relatedness of psychological constructs. Further prognostic research needs to take the interaction and moderation effect of these psychological factors into account when interpreting their results [14, 36].

Based on our findings a biopsychosocial view on patients with nonspecific acute- and subacute, non-traumatic, neck pain seems to be important. It is known that physiotherapists only partially recognize the need to address the psychosocial obstacles to recovery [37, 38]. Some of these factors are considered to be modifiable by physiotherapy intervention. It is known that physiotherapists feel often unprepared to treat these obstacles [38]. Consequently, whether these factors are modifiable will strongly depend on the skills of the physiotherapist. Therefore, there is a need for adequate education in the knowledge of assessing and acquiring treatment skills to incorporate the psychosocial domain in patient care [39].

## Conclusion

Following an expert meeting (m-NGT) and a two-round Delphi survey, the expert panel reached consensus (> 70%) on 26 factors. Twenty-one out of these 26 factors were found to be modifiable by the experts participating in the Delphi. Most of these factors were psychological factors.

## Supplementary information

**Additional file 1: Appendix 1.** Delphi Questionnaire round 1. **Appendix 2:** Delphi Questionnaire round 2.

## Data Availability

The datasets used and analyzed during the current study are available from the corresponding author on reasonable request.

## Abbreviations

m-NGT: Modified Nominal Group Technique
NVMETC: ‘Nederlandse Vereniging voor Medische Ethische Toetsingscommissies’ (In English: Dutch Association of Medical Research Ethics Committees)
MV: Martine Verwoerd
HW: Harriet Wittink
FM: Francois Maissan
ER: Edwin de Raaij
RS: Rob Smeets
