# Supplementary material for: Consensus of potential modifiable prognostic factors for persistent pain after a first episode of nonspecific idiopathic, non-traumatic neck pain: results of nominal group and Delphi technique approach
Source: BMC Musculoskelet Disord. 2020 Oct 7;21:656. doi: 10.1186/s12891-020-03682-8 (PMC7541283; doi:10.1186/s12891-020-03682-8)
Supplement: Supplementary file 1 — Additional file 1: Appendix 1. Delphi Questionnaire round 1. Appendix 2: Delphi Questionnaire round 2. [file 12891_2020_3682_MOESM1_ESM.docx]

**Appendix 1: Delphi Questionnaire round 1**

| **Prognostic factors for persistent pain after a first episode of nonspecific idiopathic, non-traumatic neck pain.  A Delphi Study** |
| --- |


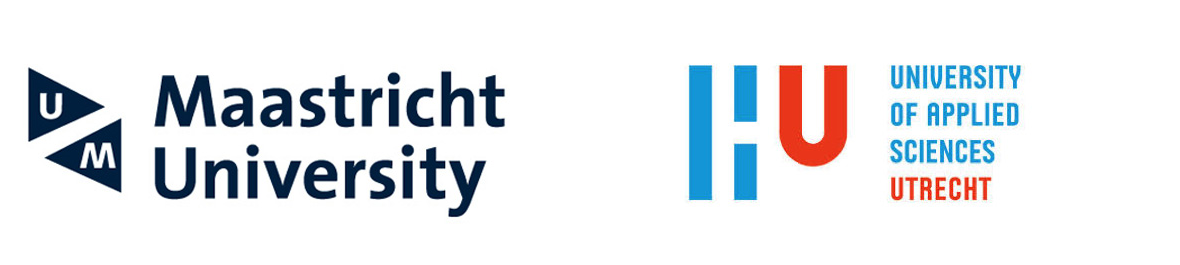


**Prognostic factors for persistent pain after a first episode of nonspecific idiopathic, non-traumatic neck pain.

A Delphi Study**
 
We would like to invite you to participate in a Delphi study at the University of Applied Sciences Utrecht in collaboration with Maastricht University (department Rehabilitation Medicine).

We recently conducted a systematic review of the literature regarding prognostic factors of acute- and subacute, non-traumatic, neck pain for chronification. The review reveals that it is still unclear which factors are prognostic for chronification of acute- and subacute neck pain. To prevent chronic neck pain, and to specifically intervene on modifiable prognostic factors, more research is important.

We are therefore starting a longitudinal prognostic cohort study to acquire more knowledge in this area. Within this study, we will focus on prognostic factors that are modifiable by a physiotherapist; we will not focus on risk factors or predictive factors (see below for our definitions).

To create a list of potential and relevant prognostic factors, we would like to invite you to participate as an expert in this Delphi Study. We have invited scientists, practicing GP’s, physiotherapists with different specializations, remedial therapists and other health care providers to participate in the Delphi Study.  

The design of our Delphi Study is as follows. In the first round, and based on your input, we will send out a questionnaire to experts in the field. The responses will then be analyzed. In the second round, and based on our analysis, we will send out a follow-up questionnaire. This procedure will be repeated until consensus is reached, or until no new information emerges. The questionnaire will consist of a number of open and a number of closed questions.

We would be very grateful if you could find some time to participate in this Delphi Study. Completing this questionnaire takes around 10-15 minutes. 

If you have any questions or want further information, please do not hesitate to contact Martine Verwoerd (martine.verwoerd@hu.nl).
 
Thank you very much for your time.
 
Yours sincerely,
 
Martine Verwoerd PT
Harriet Wittink PhD PT
Francois Maissan MSc
Professor Rob Smeets PhD MD

 
“Definitions”:

Prognostic factors are factors that influence the natural course of a disease in patients. Prognostic factors concern patients that suffer from acute- or subacute neck pain, and who need care for their acute- or subacute, non-traumatic, neck pain by a physiotherapist.

Predictive factors are defined as characteristics that identify subgroups of treated patients having different outcomes and can be used to help predict whether a person’s neck pain will respond to a specific treatment. Before clinical characteristics can be used to justify specific treatments, it is imperative that the prognostic effects of these characteristics are distinguished from their ability to predict a differential clinical benefit from a specific treatment.

Risk factors are factors that increase the risk for developing a disease. Risk factors concern people who do not suffer from acute- or subacute neck pain yet, and who therefore do not need care by a physiotherapist yet. In our study, we are not looking for risk factors.

Acute-, subacute and chronic pain are conform the definition of the ICD-11: acute pain has a duration of 0-2 weeks; subacute pain has a duration of 2 weeks to 3 months; chronic pain has a duration of more than 3 months, or is pain with a recurrent character.

We guarantee that your answers are treated completely anonymous.

To allow follow-up questions and to get some demographic statistics about the respondents of our study, please provide us with some personal details.

Name:

|  |
| --- |

E-mail address:

|  |
| --- |

Country:

|  |
| --- |

Specialisation or expertise:

|  |
| --- |

We have identified 12 different categories of prognostic factors. In the following pages, we will present you a list of factors separately for each category. We start with the "social demographic" category on this page. 

Please indicate for each factor whether you consider this factor as a prognostic factor for chronification in patients with acute- or subacute, non-traumatic, neck pain.
In case you consider a factor as prognostic, please also indicate whether you expect this factor to be modifiable by a physiotherapeutic intervention and how we should measure the factor in practice.

If you think that some prognostic factors are missing on our lists, please write them down under the bullet point "Alternative". 

To give an example:
Prior research shows that low back pain at the start of the first episode of neck pain is a prognostic factor for chronification of the neck pain. Please ask yourself whether indeed lower back pain is the prognostic factor, or whether there is an underlying factor (or factors) that has an impact on both low back pain and neck pain.

**Category: Social demographic**

|  |
| --- |

Gender

|  |
| --- |

Age

|  |
| --- |

Social class

|  |
| --- |

Education level

|  |
| --- |

Marital status

|  |
| --- |

Alternative:

|  |
| --- |

|  |
| --- |

|  |
| --- |

Is 'your alternative' modifiable? Yes No

How would you measure this factor in clinical practice?

|  |
| --- |

Please indicate for each factor whether you consider this factor as a prognostic factor for chronification in patients with acute- or subacute, non-traumatic, neck pain.

In case you consider a factor as prognostic, please indicate whether you expect this factor to be modifiable by a physiotherapeutic intervention and how we should measure the factor in practice.

**Category: Work-related factors**

|  |
| --- |

Employment status

|  |
| --- |

Happiness in work

|  |
| --- |

Physical work

|  |
| --- |

Alternative:

|  |
| --- |

| Yes |  | No |  |
| --- | --- | --- | --- |

Is 'Employment status' modifiable?

How would you measure this factor in clinical practice?

|  |
| --- |

| Yes |  | No |  |
| --- | --- | --- | --- |

Is 'Happiness in work' modifiable?

How would you measure this factor in clinical practice?

|  |
| --- |

| Yes |  | No |  |
| --- | --- | --- | --- |

Is 'Physical work' modifiable?

How would you measure this factor in clinical practice?

|  |
| --- |

| Yes |  | No |  |
| --- | --- | --- | --- |

Is 'your alternative' modifiable?

How would you measure this factor in clinical practice?

|  |
| --- |

Please indicate for each factor whether you consider this factor as a prognostic factor for chronification in patients with acute- or subacute, non-traumatic, neck pain.

In case you consider a factor as prognostic, please indicate whether you expect this factor to be modifiable by a physiotherapeutic intervention and how we should measure the factor in practice.

**Category: Symptoms**

|  |
| --- |

Pain intensity at baseline

|  |
| --- |

Duration of the neck pain

|  |
| --- |

Disturbed sleep due to neck pain

|  |
| --- |

Reported pain in different body regions

|  |
| --- |

High severity of disability

|  |
| --- |

High severity of experienced disability (ability)

|  |
| --- |

Cervical mobility

|  |
| --- |

Thoracic mobility

|  |
| --- |

Cervical motor control

|  |
| --- |

Posture

|  |
| --- |

Radiating pain below elbow

|  |
| --- |

Accompanying headache

|  |
| --- |

Dizzyness

|  |
| --- |

Pressure sensitivity neck musculature

|  |
| --- |

Alternative:

|  |
| --- |

| Yes |  | No |  |
| --- | --- | --- | --- |

Is ‘pain intensity at baseline modifiable?’

How would you measure this factor in clinical practice?

|  |
| --- |

| Yes |  | No |  |
| --- | --- | --- | --- |

Is 'disturbed sleep due to neck pain' modifiable?

How would you measure this factor in clinical practice?

|  |
| --- |

| Yes |  | No |  |
| --- | --- | --- | --- |

Is 'high severity of disability' modifiable?

How would you measure this factor in clinical practice?

|  |
| --- |

| Yes |  | No |  |
| --- | --- | --- | --- |

Is 'high severity of experienced disability' modifiable?

How would you measure this factor in clinical practice?

|  |
| --- |

| Yes |  | No |  |
| --- | --- | --- | --- |

Is 'cervical mobility' modifiable?

How would you measure this factor in clinical practice?

|  |
| --- |

| Yes |  | No |  |
| --- | --- | --- | --- |

Is 'cervical motor control' modifiable?

How would you measure this factor in clinical practice?

|  |
| --- |

| Yes |  | No |  |
| --- | --- | --- | --- |

Is 'posture' modifiable?

How would you measure this factor in clinical practice?

|  |
| --- |

| Yes |  | No |  |
| --- | --- | --- | --- |

Is 'radiating pain below elbow' modifiable?

How would you measure this factor in clinical practice?

|  |
| --- |

| Yes |  | No |  |
| --- | --- | --- | --- |

Is 'accompanying headache' modifiable?

How would you measure this factor in clinical practice?

|  |
| --- |

| Yes |  | No |  |
| --- | --- | --- | --- |

Is 'dizzyness' modifiable?

How would you measure this factor in clinical practice?

|  |
| --- |

| Yes |  | No |  |
| --- | --- | --- | --- |

Is 'pressure sensitivity neck musculature' modifiable?

How would you measure this factor in clinical practice?

|  |
| --- |

| Yes |  | No |  |
| --- | --- | --- | --- |

Is 'your alternative' modifiable?

How would you measure this factor in clinical practice?

|  |
| --- |

Please indicate for each factor whether you consider this factor as a prognostic factor for chronification in patients with acute- or subacute, non-traumatic, neck pain.

In case you consider a factor as prognostic, please indicate whether you expect this factor to be modifiable by a physiotherapeutic intervention and how we should measure the factor in practice.

**Category: Prior conditions**

Neck pain before

History of musculoskeletal pain

Alternative:

|  |
| --- |

| Yes |  | No |  |
| --- | --- | --- | --- |

Is 'your alternative' modifiable?

How would you measure this factor in clinical practice?

|  |
| --- |

Please indicate for each factor whether you consider this factor as a prognostic factor for chronification in patients with acute- or subacute, non-traumatic, neck pain.

In case you consider a factor as prognostic, please indicate whether you expect this factor to be modifiable by a physiotherapeutic intervention and how we should measure the factor in practice.

**Category: General health**

|  |
| --- |

Physical inactivity

|  |
| --- |

Unhealthy lifestyle (smoking, alcohol, eating etc.)

|  |
| --- |

Sleep quality

|  |
| --- |

Alternative:

|  |
| --- |

| Yes |  | No |  |
| --- | --- | --- | --- |

Is 'physical inactivity' modifiable?

How would you measure this factor in clinical practice?

|  |
| --- |

| Yes |  | No |  |
| --- | --- | --- | --- |

Is 'unhealthy lifestyle' modifiable?

How would you measure this factor in clinical practice?

|  |
| --- |

| Yes |  | No |  |
| --- | --- | --- | --- |

Is 'sleep quality' modifiable?

How would you measure this factor in clinical practice?

|  |
| --- |

| Yes |  | No |  |
| --- | --- | --- | --- |

Is 'your alternative' modifiable?

How would you measure this factor in clinical practice?

|  |
| --- |

The following categories focus more on **psychological factors**. Since assigning factors to a specific "psychological" category involves judgement, please indicate in the following text box whether you think that a certain factor better belongs to another category.

For your overview, here is the list of all categories that focus on psychological factors:
Cognition, Emotions, Behavior, Perceptions, Motivation, Vulnerability.

|  |
| --- |

Please indicate for each factor whether you consider this factor as a prognostic factor for chronification in patients with acute- or subacute, non-traumatic, neck pain.

In case you consider a factor as prognostic, please indicate whether you expect this factor to be modifiable by a physiotherapeutic intervention and how we should measure the factor in practice.

**Category: Cognition**

|  |
| --- |

Somatization

|  |
| --- |

Catastrophizing

|  |
| --- |

Locus of control

|  |
| --- |

Acceptance of illness

|  |
| --- |

Illness beliefs about recovery

|  |
| --- |

Treatment beliefs

|  |
| --- |

Alternative:

|  |
| --- |

*Explanation prognostic factors

Somatization: when physical symptoms are caused by psychological or emotional factors.
Catastrophizing: an exaggerated negative orientation towards a negative stimuli. This makes catastrophizing a cognitive phenomenon.
Locus of control: this can be internal or external control. Internal control: the extent to which the patient thinks he/she can control the pain. External control: the extent to which the patient thinks that other people can control his/her pain.
Treatment beliefs: this includes the patient's beliefs about how treatment may help to control or recover from the illness.*

| Yes |  | No |  |
| --- | --- | --- | --- |

Is 'somatization' modifiable?

How would you measure this factor in clinical practice?

|  |
| --- |

| Yes |  | No |  |
| --- | --- | --- | --- |

Is 'catastrophizing' modifiable?

How would you measure this factor in clinical practice?

|  |
| --- |

| Yes |  | No |  |
| --- | --- | --- | --- |

Is 'locus of control' modifiable?

How would you measure this factor in clinical practice?

|  |
| --- |

| Yes |  | No |  |
| --- | --- | --- | --- |

Is 'acceptance of illness' modifiable?

How would you measure this factor in clinical practice?

|  |
| --- |

| Yes |  | No |  |
| --- | --- | --- | --- |

Is 'illness beliefs about recovery' modifiable?

How would you measure this factor in clinical practice?

|  |
| --- |

| Yes |  | No |  |
| --- | --- | --- | --- |

Is 'treatment beliefs' modifiable?

How would you measure this factor in clinical practice?

|  |
| --- |

| Yes |  | No |  |
| --- | --- | --- | --- |

Is 'your alternative' modifiable?

How would you measure this factor in clinical practice?

|  |
| --- |

The following categories focus more on **psychological factors**. Since assigning factors to a specific "psychological" category involves judgement, please indicate in the following text box whether you think that a certain factor better belongs to another category. 

For your overview, here is the list of all categories that focus on psychological factors:
Cognition, Emotions, Behavior, Perceptions, Motivation, Vulnerability.

|  |
| --- |

Please indicate for each factor whether you consider this factor as a prognostic factor for chronification in patients with acute- or subacute, non-traumatic, neck pain.

In case you consider a factor as prognostic, please indicate whether you expect this factor to be modifiable by a physiotherapeutic intervention and how we should measure the factor in practice.

**Category: Emotions**

|  |
| --- |

Depression

|  |
| --- |

Kinesiophobia

|  |
| --- |

Distress

|  |
| --- |

Anger

|  |
| --- |

Injustice

|  |
| --- |

Alternative:

|  |
| --- |

*Explanation prognostic factors

Depression: condition that comes under 'mental illness'. There is a depressive mood when there is an abnormal depression for a longer period (longer than two weeks) and/or an abnormal lethargy, loss of interest or an inability to enjoy something. We mean both light and heavier depressions. 
Kinesiophobia: fear of movement.
Distress: negative stress. This means stress that is not in the interests of a person and is experiences as a unpleasant external stimulus.
Anger: angry mood (irritability, frustration) or a negative social cognitions (interpersonal sensitivity, envy, disagreeableness).*

| Yes |  | No |  |
| --- | --- | --- | --- |

Is 'depression' modifiable?

How would you measure this factor in clinical practice?

|  |
| --- |

| Yes |  | No |  |
| --- | --- | --- | --- |

Is 'kinesiophobia' modifiable?

How would you measure this factor in clinical practice?

|  |
| --- |

| Yes |  | No |  |
| --- | --- | --- | --- |

Is 'distress' modifiable?

How would you measure this factor in clinical practice?

|  |
| --- |

| Yes |  | No |  |
| --- | --- | --- | --- |

Is 'anger' modifiable?

How would you measure this factor in clinical practice?

|  |
| --- |

| Yes |  | No |  |
| --- | --- | --- | --- |

Is 'injustice' modifiable?

How would you measure this factor in clinical practice?

|  |
| --- |

| Yes |  | No |  |
| --- | --- | --- | --- |

Is 'your alternative' modifiable?

How would you measure this factor in clinical practice?

|  |
| --- |

The following categories focus more on **psychological factors**. Since assigning factors to a specific "psychological" category involves judgement, please indicate in the following text box whether you think that a certain factor better belongs to another category. 

For your overwiev, here is the list of all categories that focus on psychological factors:
Cognition, Emotions, Behavior, Perceptions, Motivation, Vulnerability

|  |
| --- |

Please indicate for each factor whether you consider this factor as a prognostic factor for chronification in patients with acute- or subacute, non-traumatic, neck pain.

In case you consider a factor as prognostic, please indicate whether you expect this factor to be modifiable by a physiotherapeutic intervention and how we should measure the factor in practice.

**Category: Behavior**

|  |
| --- |

Coping

|  |
| --- |

Alternative:

|  |
| --- |

*Explanation prognostic factors

Coping: the way someone deals with problems or stress.*

| Yes |  | No |  |
| --- | --- | --- | --- |

Is 'coping' modifiable?

How would you measure this factor in clinical practice?

|  |
| --- |

| Yes |  | No |  |
| --- | --- | --- | --- |

Is 'your alternative' modifiable?

How would you measure this factor in clinical practice?

|  |
| --- |

The following categories focus more on **psychological factors**. Since assigning factors to a specific "psychological" category involves judgement, please indicate in the following text box whether you think that a certain factor better belongs to another category. 

For your overview, here is the list of all categories that focus on psychological factors:
Cognition, Emotions, Behavior, Perceptions, Motivation, Vulnerability.

|  |
| --- |

Please indicate for each factor whether you consider this factor as a prognostic factor for chronification in patients with acute- or subacute, non-traumatic, neck pain.

In case you consider a factor as prognostic, please indicate whether you expect this factor to be modifiable by a physiotherapeutic intervention and how we should measure the factor in practice.

**Category: Perceptions**

|  |
| --- |

Illness beliefs about pain identity

|  |
| --- |

Hypervigilance

|  |
| --- |

Alternative:

|  |
| --- |

*Explanation prognostic factors

Illness beliefs about pain identity: This includes perceptions about the label or name given to the condition by patients and the symptoms that are perceived to go with it.
Hypervigilance: to pain or somatic sensations is the excessive tendency to attend to pain/somatic sensations, or the excessive readiness to select pain-related information over other information from the environment.*

| Yes |  | No |  |
| --- | --- | --- | --- |

Is 'illness beliefs about pain identity' modifiable?

How would you measure this factor in clinical practice?

|  |
| --- |

| Yes |  | No |  |
| --- | --- | --- | --- |

Is 'hypervigilance' modifiable?

How would you measure this factor in clinical practice?

|  |
| --- |

| Yes |  | No |  |
| --- | --- | --- | --- |

Is 'your alternative' modifiable?

How would you measure this factor in clinical practice?

|  |
| --- |

The following categories focus more on **psychological factors**. Since assigning factors to a specific "psychological" category involves judgement, please indicate in the following text box whether you think that a certain factor better belongs to another category. 

For your overview, here is the list of all categories that focus on psychological factors:
Cognition, Emotions, Behavior, Perceptions, Motivation, Vulnerability.

|  |
| --- |

Please indicate for each factor whether you consider this factor as a prognostic factor for chronification in patients with acute- or subacute, non-traumatic, neck pain.

In case you consider a factor as prognostic, please indicate whether you expect this factor to be modifiable by a physiotherapeutic intervention and how we should measure the factor in practice.

**Category: Motivation**

|  |
| --- |

Purposeful behavior

|  |
| --- |

Alternative:

|  |
| --- |

| Yes |  | No |  |
| --- | --- | --- | --- |

Is 'purposeful behavior' modifiable?

How would you measure this factor in clinical practice?

|  |
| --- |

| Yes |  | No |  |
| --- | --- | --- | --- |

Is 'your alternative' modifiable?

How would you measure this factor in clinical practice?

|  |
| --- |

The following categories focus more on **psychological factors**. Since assigning factors to a specific "psychological" category involves judgement, please indicate in the following text box whether you think that a certain factor better belongs to another category.

For your overview, here is the list of all categories that focus on psychological factors:
Cognition, Emotions, Behavior, Perceptions, Motivation, Vulnerability.

Please indicate for each factor whether you consider this factor as a prognostic factor for chronification in patients with acute- or subacute, non-traumatic, neck pain.

In case you consider a factor as prognostic, please indicate whether you expect this factor to be modifiable by a physiotherapeutic intervention and how we should measure the factor in practice.

**Category: Vulnerability**

|  |
| --- |

Limited health literacy

|  |
| --- |

Limited self-regulation

|  |
| --- |

Limited self-efficacy

|  |
| --- |

Alternative:

|  |
| --- |

*Explanation prognostic factors

Health literacy: has been defined as the cognitive and social skills which determine the motivation and ability of individuals to gain access to, understand and use information in ways which promote and maintain good health.
Self-regulation: a plan for patients to eliminate health risk behaviors. It includes self-monitoring, self-evaluation, and self-reinforcement.
Self-efficacy: confidence in ability to successfully perform specific tasks or behaviors related to one's health in a variety of situations.*

| Yes |  | No |  |
| --- | --- | --- | --- |

Is 'limited health literacy' modifiable?

How would you measure this factor in clinical practice?

|  |
| --- |

| Yes |  | No |  |
| --- | --- | --- | --- |

Is 'limited self-regulation' modifiable?

How would you measure this factor in clinical practice?

|  |
| --- |

| Yes |  | No |  |
| --- | --- | --- | --- |

Is 'limited self-efficacy' modifiable?

How would you measure this factor in clinical practice?

|  |
| --- |

| Yes |  | No |  |
| --- | --- | --- | --- |

Is 'your alternative' modifiable?

How would you measure this factor in clinical practice?

|  |
| --- |

**Remaining factors**

|  |
| --- |

Health care provider attitude (biomedical/ biopsychosocial)

|  |
| --- |

Therapeutic relation

|  |
| --- |

Alternative:

|  |
| --- |

| Yes |  | No |  |
| --- | --- | --- | --- |

Is 'health care provider attitude' modifiable?

How would you measure this factor in clinical practice?

|  |
| --- |

| Yes |  | No |  |
| --- | --- | --- | --- |

Is 'therapeutic relation' modifiable?

How would you measure this factor in clinical practice?

|  |
| --- |

| Yes |  | No |  |
| --- | --- | --- | --- |

Is 'your alternative' modifiable?

How would you measure this factor in clinical practice?

|  |
| --- |

Thank you very much for your participation! We highly appreciate it.

We will send you the results of this first round of our Delphi study. We will also ask you again to participate in the second round of our Delphi study.

If there is anything you would like us to know, please use the comment field below.

|  |
| --- |

**Appendix 2: Delphi Questionnaire round 2**

**Prognostic factors for persistent pain after a first episode of nonspecific idiopathic, non-traumatic neck pain.

A Delphi Study - Round 2**


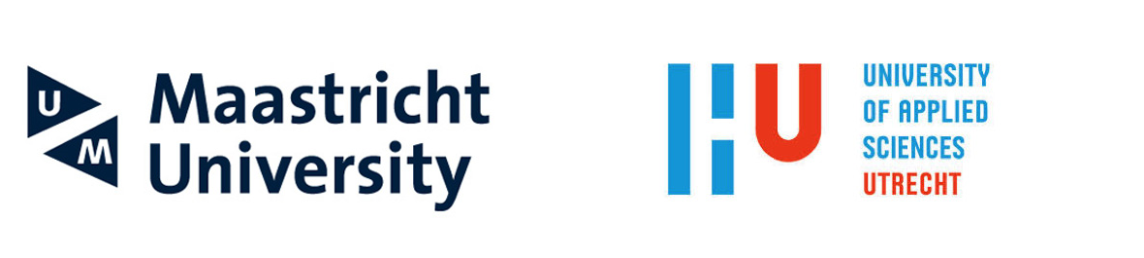


**Prognostic factors for persistent pain after a first episode of nonspecific idiopathic, non-traumatic neck pain.

Second and final round Delphi Study**

First of all, we are very thankful that nearly 90 experts participated in the first round of our Delphi Study. We very much appreciate your opinion and feedback. **You can find the results of the first round below.**
 
We now invite you to participate in the second and final round of our Delphi study.

The goal is to obtain a list of potential modifiable prognostic factors for chronification of acute- and subacute neck pain (please see our definitions of terms below). This final list of factors will form the basis of the subsequent longitudinal prognostic cohort study. 

The second questionnaire is significantly shorter than the first one and consits of some open and closed questions. It will take approximately 5 to 10 minutes of your time.  

We would again be very grateful if you can spare some time and also participate in the final round of our Delphi Study.

If you have any questions or want further information, please do not hesitate to contact Martine Verwoerd (martine.verwoerd@hu.nl).
 
Thank you very much for your time.
 
Yours sincerely,
 
Martine Verwoerd PT
Harriet Wittink PhD PT
Francois Maissan MSc
Professor Rob Smeets PhD MD

“Definitions”:

*Acute-, subacute and chronic pain*are conform the definition of the ICD-11.

*Acute pain*has a duration of 0-2 weeks;
*Subacute pain*has a duration of 2 weeks to 3 months;
*Chronic pain*has a duration of more than 3 months, or is pain with a recurrent character.

We guarantee that your answers are treated completely anonymous.

To allow follow-up questions and to get some demographic statistics about the respondents of our study, please provide us with some personal details.

Name:

|  |
| --- |

E-mail address:

|  |
| --- |

Country:

|  |
| --- |

Specialisation or expertise:

|  |
| --- |

**Second round Delphi - Part 1: Reconcider prognostic factors**

There were some prognostic factors with an agreement score between 60-70%. We would like to get more input on these factors.

Prior literature considers some of the factors as prognostic for musculoskeletal pain in **other regions**. To avoid false-negative findings on such factors, can you please indicate (again) whether you consider the factors as being prognostic for chronification in patients with acute- or subacute, non-traumatic **neck pain**.

If you consider this factor to be **not** prognostic for chronification of **neck pain**, can you please describe why not.
If you consider this factor to be prognostic for chronification of **neck pain**, please indicate whether you expect this factor to be modifiable by a physiotherapeutic intervention.

You find further information and definitions of our concepts by clicking on the question mark sign.

|  |
| --- |

Pain intensity at baseline

|  |
| --- |

High severity of experienced disability

|  |
| --- |

Somatization

|  |
| --- |

Limited health literacy

| Yes |  | No |  |
| --- | --- | --- | --- |

Is 'pain intensity at baseline' modifiable?

Please describe why you consider 'pain at baseline' not as a prognostic factor.

|  |
| --- |

| Yes |  | No |  |
| --- | --- | --- | --- |

Is 'experienced disability' modifiable?

Please describe why you consider 'high severity of experienced disability' not as a prognostic factor.

|  |
| --- |

| Yes |  | No |  |
| --- | --- | --- | --- |

Is 'somatization' modifiable?

Please describe why you consider 'somatization' not as a prognostic factor.

|  |
| --- |

| Yes |  | No |  |
| --- | --- | --- | --- |

Is 'limited health literacy' modifiable?

Please describe why you consider 'limited health literacy' not as a prognostic factor.

|  |
| --- |

**Second round Delphi - Part 2: Additional prognostic factors**

We present you some more potentially prognostic factors. Please indicate whether you consider each factor as a prognostic factor for chronification in patients with acute- or subacute, non-traumatic, neck pain.

If you consider a factor as prognostic, please indicate whether you expect this factor to be **modifiable** by a physiotherapeutic intervention and how we should **measure** the factor in practice.

|  |
| --- |

Orofacial pain

|  |
| --- |

Potential to self-modify posture during work

| Yes |  | No |  |
| --- | --- | --- | --- |

Is 'orofacial pain' modifiable?

How would you measure this factor in clinical practice?

|  |
| --- |

| Yes |  | No |  |
| --- | --- | --- | --- |

Is 'potential to self-modify posture during work' modifiable?

How would you measure this factor in clinical practice?

|  |
| --- |

For two prognostic factors with overall high agreement scores we have difficulties in assessing what these factors actually mean and how we could measure them.
These factors are (1) sleep quality, and (2) happiness at work. We therefore ask you for your input.

(1) **Sleep quality**: Please describe (i) what you consider to be "bad sleep quality", and (ii) how you would measure "bad sleep quality".

| (1)  (2) |
| --- |

(2) **Happiness at work**: We consider using the following question in our study to capture happiness at work:

*"On a numeric rating scale from 0 to 10, how satisfied are you in your work?* (0 = not satisfied at all, 10 = totally satisfied)"

Please tell us what comes to your mind when reading this question.

We would like you to describe in a few sentences, what you think we do exactly measure with this question?

|  |
| --- |

Thank you very much for your participation! We highly appreciate it.

We will send you the results of the second round of our Delphi study. We do not expect to have a third round. 

If there is anything you would like us to know, please use the comment field below.

|  |
| --- |
